# Supplementary material for: On the origin of elasticity and heat conduction anisotropy of liquid crystal elastomers at gigahertz frequencies
Source: Nat Commun. 2022 Sep 6;13:5248. doi: 10.1038/s41467-022-32865-1 (PMC9448779; doi:10.1038/s41467-022-32865-1)
Supplement: Supplementary file 1 — Supplementary Information [file 41467_2022_32865_MOESM1_ESM.pdf]

Supplementary Information

On the origin of elasticity and heat conduction anisotropy of liquid crystal elastomers at gigahertz frequencies

Yu Cang[1,2], Jiaqi Liu[3], Meguya Ryu[4,5], Bartlomiej Graczykowski[2,6], Junko Morikawa[4], Shu Yang[3]*, George Fytas[2]*

[1]School of Aerospace Engineering and Applied Mechanics, Tongji University, Zhangwu Road 100, Shanghai 200092, China
[2]Max Planck Institute for Polymer Research, Ackermannweg 10, Mainz 55128, Germany
[3]Department of Materials Science and Engineering, University of Pennsylvania, 3231 Walnut Street, Philadelphia, PA 19104, USA
[4]School of Materials and Chemical Technology, Tokyo Institute of Technology, Ookayama, Meguro-ku, Tokyo 152-8550, Japan
[5]National Metrology Institute of Japan (NMIJ) National Institute of Advanced Industrial Science and Technology (AIST), Umezono, Tsukuba 305-8563, Japan
[6]Faculty of Physics, Adam Mickiewicz University, Uniwersytetu Poznanskiego 2, Poznan 61-614, Poland

*Corresponding authors: fytas@mpip-mainz.mpg.de; shuyang@seas.upenn.edu

Contents

1. **Supplementary Section 1. Refractive index;**
2. **Supplementary Section 2. Viscosity coefficients;**
3. **Supplementary Table 1-3;**
4. **Supplementary Figure 1-12.**

Supplementary Section 1. Refractive index

The refractive index n is estimated from the dispersion relation $f(q)$ of acoustic phonon, where the frequency f is obtained from the BLS spectra at a given q. The n enters to the expression of $q(= 4\pi\sqrt{n^2 - \sin^2\beta}\,/\lambda)$ in the reflection and backscattering geometries, where β is the incident angle outside the sample. Here we employed three different scattering geometries (i.e., transmission, reflection and backscattering) in the experiments to turn the magnitude of q in a wide range. Given the relationship of $f(=cq/2\pi)$ with c being sound velocity, the n is determined by forcing the q to be linear with f and $f(q)$ being through the original point (0,0). At specifically backscattering geometry with β=0°,

For the monodomain LCE, the ordinary refractive index n_o=1.72 is obtained from the $f(q)$ of Q-L phonon with **q** being normal to director **n** in Supplementary Fig. S2a. At specific backscattering geometry with β=0°, the expression of $q(= 4\pi n/\lambda)$ is simplified depending on only the n. The light senses the n_o when its polarization is normal to the optical axis (director), whereas experience of n_e as being normal to the director. Experimentally, in VV configuration, the polarization of light is set to be normal to the director, and thus is governed by n_o, while n_e enters in the HH configuration. From the Fig. 2b, f_{VV}/f_{HH}=1.10 is obtained for the same Q-L phonon propagating normal to the director. Given $f_{VV}/q_{VV}= f_{HH}/q_{HH}$ and n_o=1.72, we could obtain n_e=1.56.

The refractive index is isotropic for the polydomain LCE, which is confirmed by the polarization-independent frequency in Supplementary Fig. S4a. The n=1.63 is estimated from the dispersion relation of longitudinal and transverse acoustic phonons in Supplementary Fig. S4b.

Supplementary Section 2. Viscosity coefficients

The ϕ-dependent sound attenuation $\mu(=\pi\Gamma/c)$ is given by:

$$\mu_{Q-L} = \frac{\pi\Gamma}{c_{Q-L}} = \frac{\omega^2}{2\rho c^3}(\eta_{11}\sin^2\phi + \eta_{33}\cos^2\phi + (2\eta_{13} + 4\eta_{44} - \eta_{11} - \eta_{33})\sin^2\phi\cos^2\phi) \quad \text{(S1a)}$$

$$\alpha_{Q-T} = \frac{\pi\Gamma}{c_{Q-T}} = \frac{\omega^2}{2\rho c^3}(\eta_{44} - (2\eta_{13} + 4\eta_{44} - \eta_{11} - \eta_{33})\sin^2\phi\cos^2\phi) \quad \text{(S1b)}$$

where Γ is the linewidth (FWHM) of the BLS peak.

Given $c = \frac{\omega}{q} = \frac{2\pi f}{q}$, Equation 2-1 and 2-2 could be simplified as

$$\Gamma_{\mathrm{Q\_L}} = \frac{q^2}{2\pi\rho}(\eta_{11}\sin^2\phi + \eta_{33}\cos^2\phi + (2\eta_{13} + 4\eta_{44} - \eta_{11} - \eta_{33})\sin^2\phi\cos^2\phi) \quad \text{(S1c)}$$

$$\Gamma_{\mathrm{Q\_T}} = \frac{q^2}{2\pi\rho}(\eta_{44} - (2\eta_{13} + 4\eta_{44} - \eta_{11} - \eta_{33})\sin^2\phi\cos^2\phi) \quad \text{(S1d)}$$

Supplementary Table 1. Elastic stiffness constants (GPa) of the monodomain LCE film.

C_{11}	C_{13}	C_{33}	C_{44}	C_{66}
6.67±0.12	5.14±0.12	13.84±0.20	1.04±0.03	1.13±0.06

Supplementary Table 2. Four viscosity coefficients (0.1 Pa·s) of monodomain LCE film obtained by fitting experimental data in Fig. 2e.

η_{11}	η_{13}	η_{33}	η_{44}
0.107±0.007	-0.105±0.012	0.391±0.014	0.135±0.008

Supplementary Table 3. Engineering moduli (Young's modulus $E_{\parallel}$ and $E_{\perp}$, shear modulus G_{12} *and* G_{13}, Poisson's ratio ν_{31} and ν_{21}) of monodomain LCE, LCE-II, and Au/LCE-0.2wt% films, the plots (sound velocity vs. orientation angle) of which are shown in Fig. 2d, Supplementary Fig. S6a, and Supplementary Fig. S7a, respectively. For LCE-II and Au/LCE-0.2wt% films, we assumed $C_{44} \approx C_{66}$ and hence $G_{13} = G_{12}$.

Sample	$E_{\parallel}$ (GPa)	$E_{\perp}$ (GPa)	G_{13} (GPa)	G_{12} (GPa)	ν_{31}	ν_{12}	$E_{\parallel}/E_{\perp}$
LCE	9.07±0.21	3.44±0.13	1.04±0.02	1.13±0.06	0.46±0.01	0.53±0.03	2.63±0.13
LCE-II	9.11±0.23	3.18±0.16	1.02±0.02	1.02±0.02	0.45±0.02	0.56±0.04	2.86±0.18
Au/LCE-0.2wt%	9.24±0.18	4.16±0.12	1.50±0.02	1.5±0.02	0.486±0.01	0.388±0.02	2.22±0.11

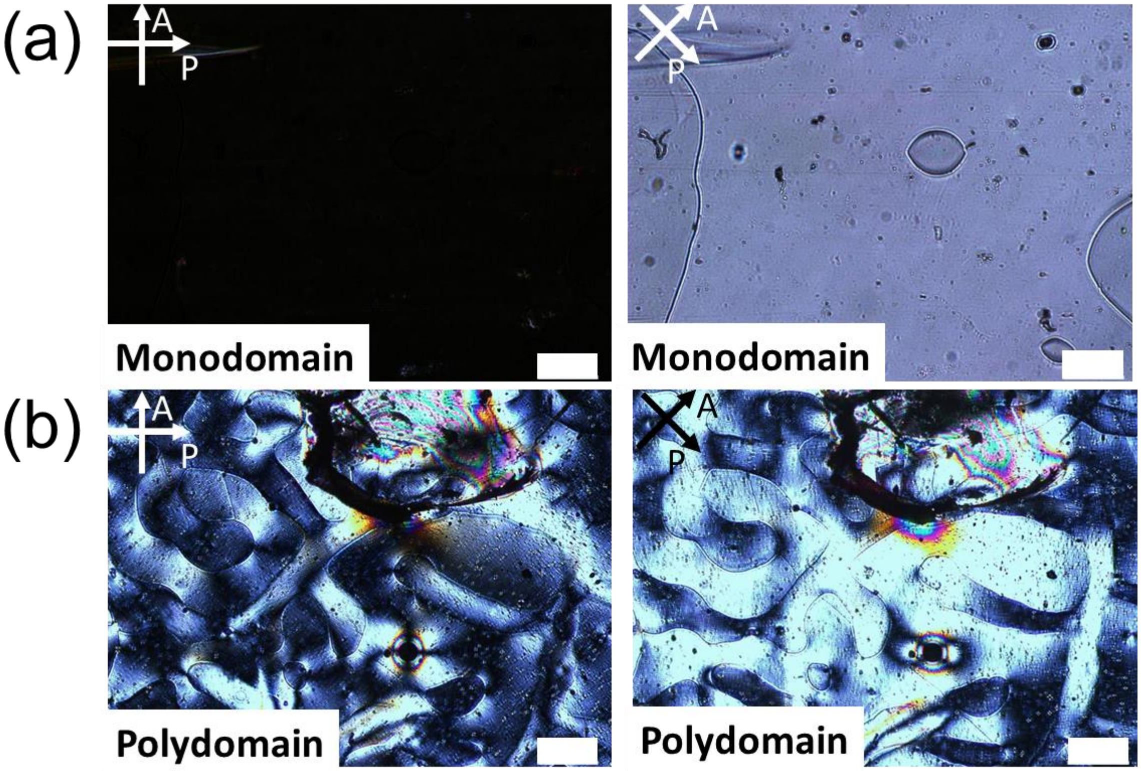

Supplementary Figure 1. Polarized optical microscopy images. (a) monodomain and (b) polydomain LCE films. In the right panel of (a) and (b), the polarizer (P) and analyzer (A) are rotated by 45°. The arrows indicate the polarization of the polarizer and analyzer. (Scale bars: 200 μm)

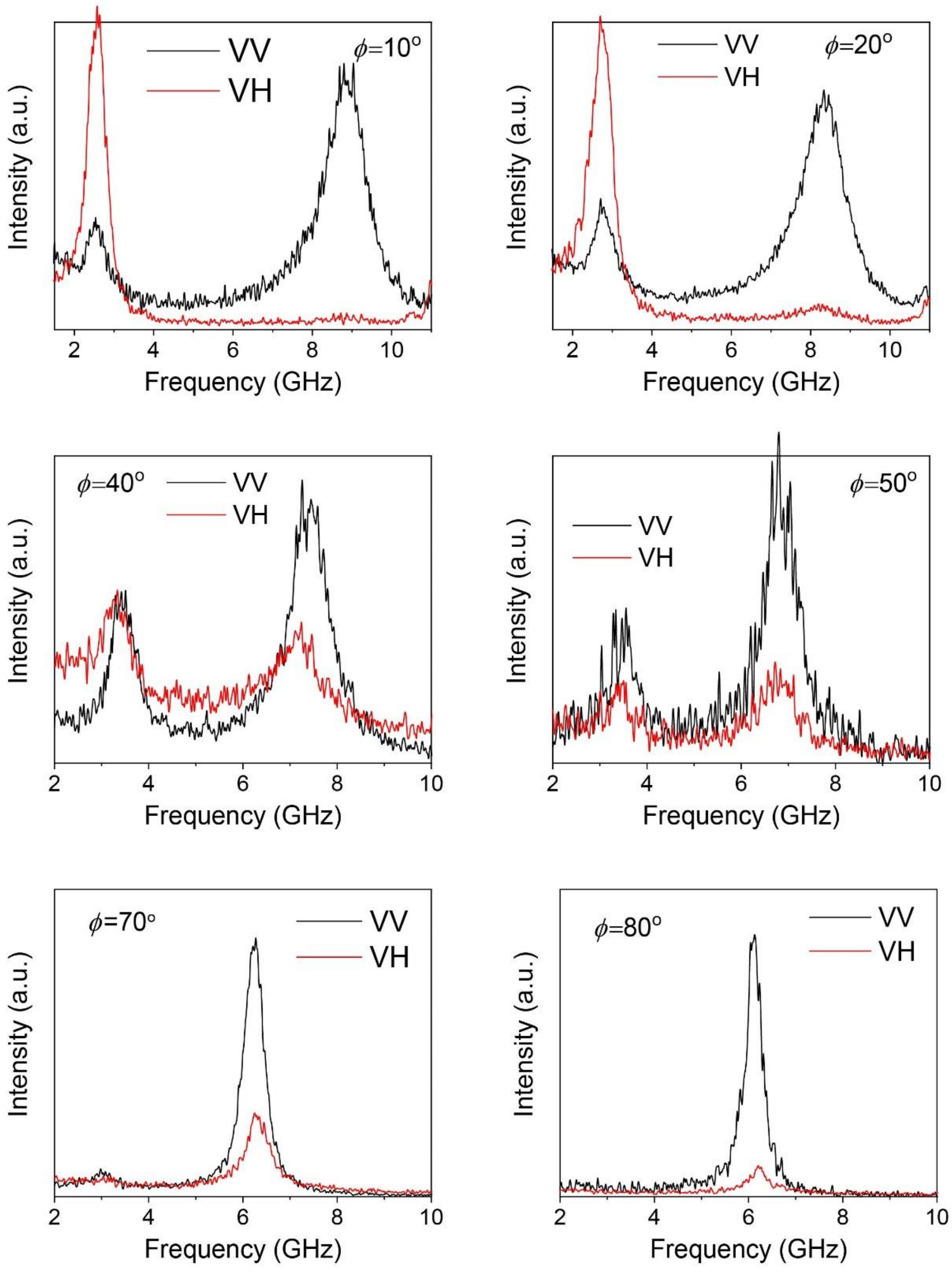

Supplementary Figure 2. VV and VH BLS spectra with different orientation angle ϕ. VV (black solid lines) and VH (red solid lines) BLS spectra are obtained at the transmission geometry, where the orientation angle ϕ varies from 10° to 80° at a given q=0.0167 nm^{-1}.

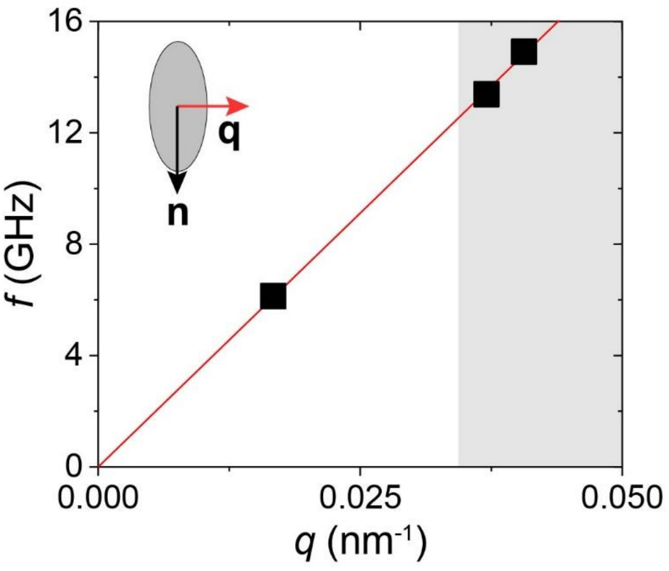

Supplementary Figure 3. Dispersion relation of quasi-longitudinal phonon with q being normal to the direction n. The $f(q)$ are fitted by linear fit (solid line), yielding n_o=1.72. The shaded area denotes the experimental data obtained from reflection and backscattering geometries.

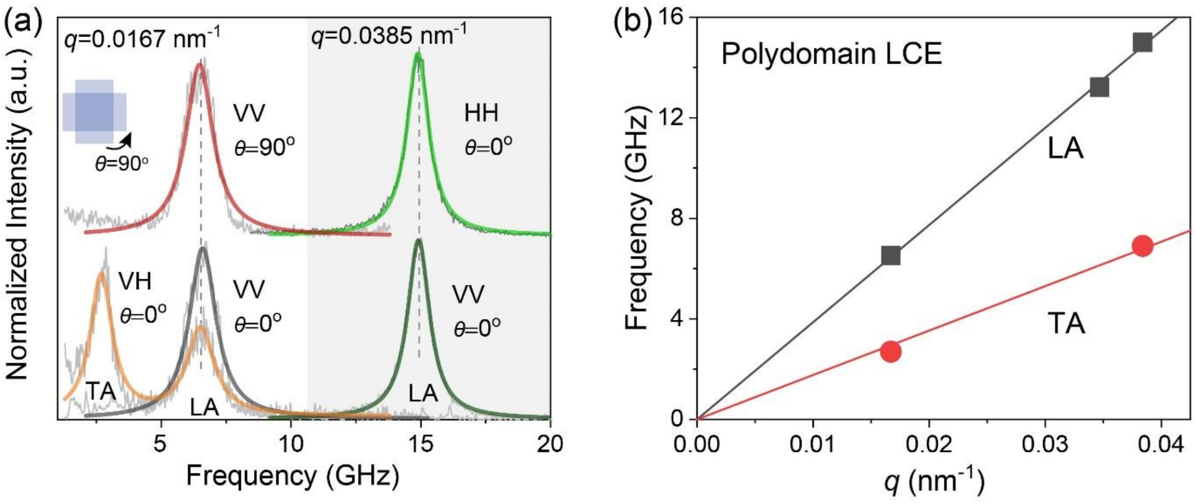

Supplementary Figure 4. BLS spectra and dispersion relation of polydomain LCE film. (a) VV and VH BLS spectra (grey lines) of the polydomain LCE film at a q=0.0167 nm^{-1}, and VV and HH BLS spectra at a q=0.0385 nm^{-1}, represented by Lorentzian shapes (colored lines). The observed single peak in polarized (VV and HH) spectra is assigned to longitudinal acoustic (LA) phonon, whereas transverse acoustic (TA) phonon is detected in the depolarized (VH) spectra. As the sample rotates from θ=0° to θ=90°, the identical frequency of LA phonon at the q=0.0167 nm^{-1} confirms the elastic isotropy. While the constant frequency of LA in different VV and HH configurations at the q=0.0385 nm^{-1} indicates the optical isotropy. The dashed lines are guide for the eye. (b) The corresponding dispersion relations of LA and TA phonons, which are fitted by linear relations (solid lines).

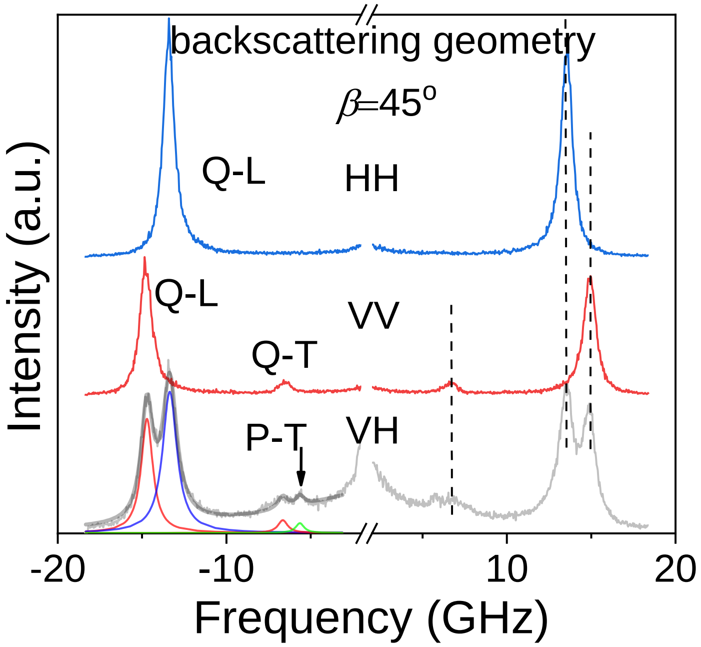

Supplementary Figure 5. VV, HH, and VH BLS spectra obtained at the backscattering geometry for monodomain LCE film. At incident angle β=45°, the Q-L and Q-T modes in VH spectra corresponds to that in the VV and HH spectra due to the birefringence. The lowest-frequency peak in VH spectra but absent in the polarized spectra can be assigned to pure transverse (P-T) mode. The VH spectra are represented by the Lorentzian shapes (thick grey line) with four individual peaks (colored lines). The dashed lines are guide to the eye. The two P-T data at high orientation angle ϕ's (Fig. 2c) allow the determination of C_{66} according to $\rho\left(\frac{2\pi}{q}\right)^2 f_{\mathrm{P-T}}^2 = \sin^2\phi\, C_{66} + \cos^2\phi\, C_{44}$ (solid line in Fig. 2c). $f_{\text{P-T}} = f_{\text{Q-T}}$ is valid when ϕ = 0°. Other four independent elastic constants (C_{11}, C_{33}, C_{13} and C_{44}) are mainly determined by $f_{\text{Q-L}}(\phi)$ and $f_{\text{Q-T}}(\phi)$.

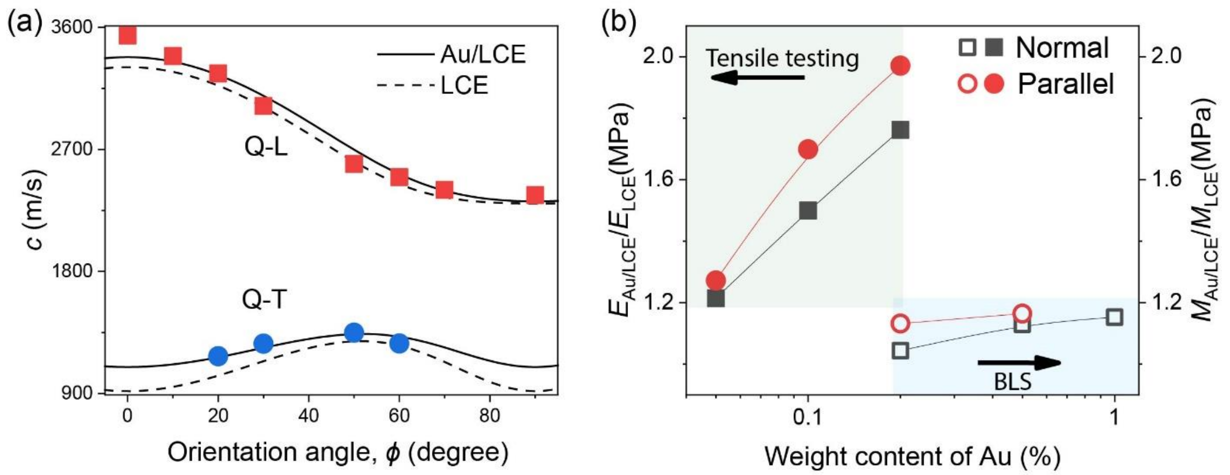

Supplementary Figure 6. Elastic moduli of Au/LCE films measured by tensile testing and BLS. (a) Quasi-longitudinal (Q-L, red squares) and quasi-transverse (Q-T, blue circles) sound velocities vs. orientation angle, ϕ, for Au/LCE with 0.2 wt% Au. The solid lines are fittings according to Christoffel's equation (Equation 2 in Methods). The dash lines are the representation of $c(\phi)$ for pure LCE obtained from Figure 2b. (b) Normalized Young's modulus $E_{\mathrm{Au/LCE}}/E_{\mathrm{LCE}}$ obtained from tensile testing[1] and normalized compression modulus $M_{\mathrm{Au/LCE}}/M_{\mathrm{LCE}}$ ($M = \rho c_{\mathrm{Q-L}}^2$) obtained from BLS, respectively, as a function of weight content of Au. Black and red symbols denote the moduli are normal and parallel to the director **n**, respectively.

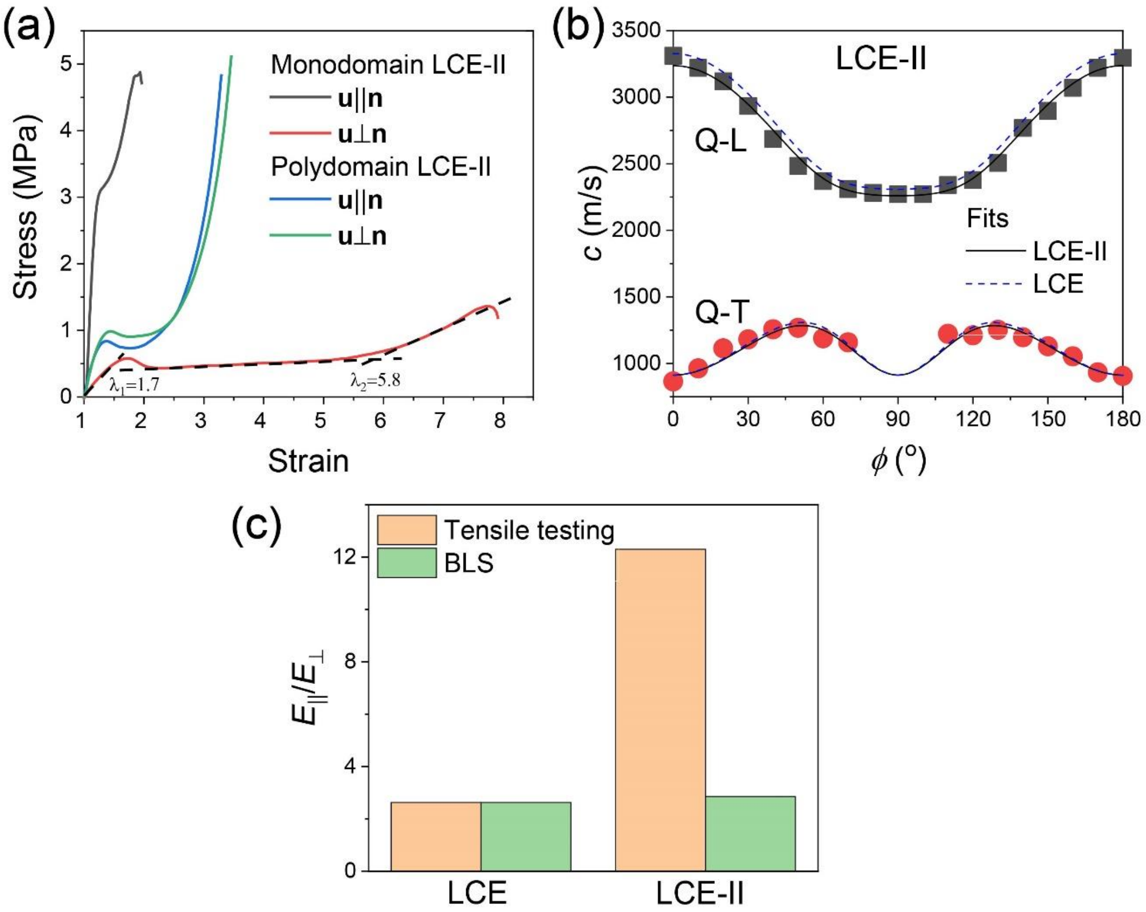

Supplementary Figure 7. Elastic anisotropy of LCE-II film measured by tensile testing and BLS. (a) Strain-stress curves of LCE-II films in monodomain and polydomain states with extension being parallel and perpendicular to the director **n**, respectively. The λ_1 and λ_2 are estimated according to literature[2,3] from intersection of dash lines representing the low-strain linear, soft-plateau, and high-strain linear regimes, respectively. (b) Sound velocities of Q-L and Q-T modes vs. orientation angle ϕ of monodomain LCE-II film. The black solid lines and blue dashed lines are fits for LCE-II and LCE respectively. (c) Comparison of Young's modulus anisotropy, $E_{||}/E_{\perp}$, obtained from tensile testing and BLS respectively.

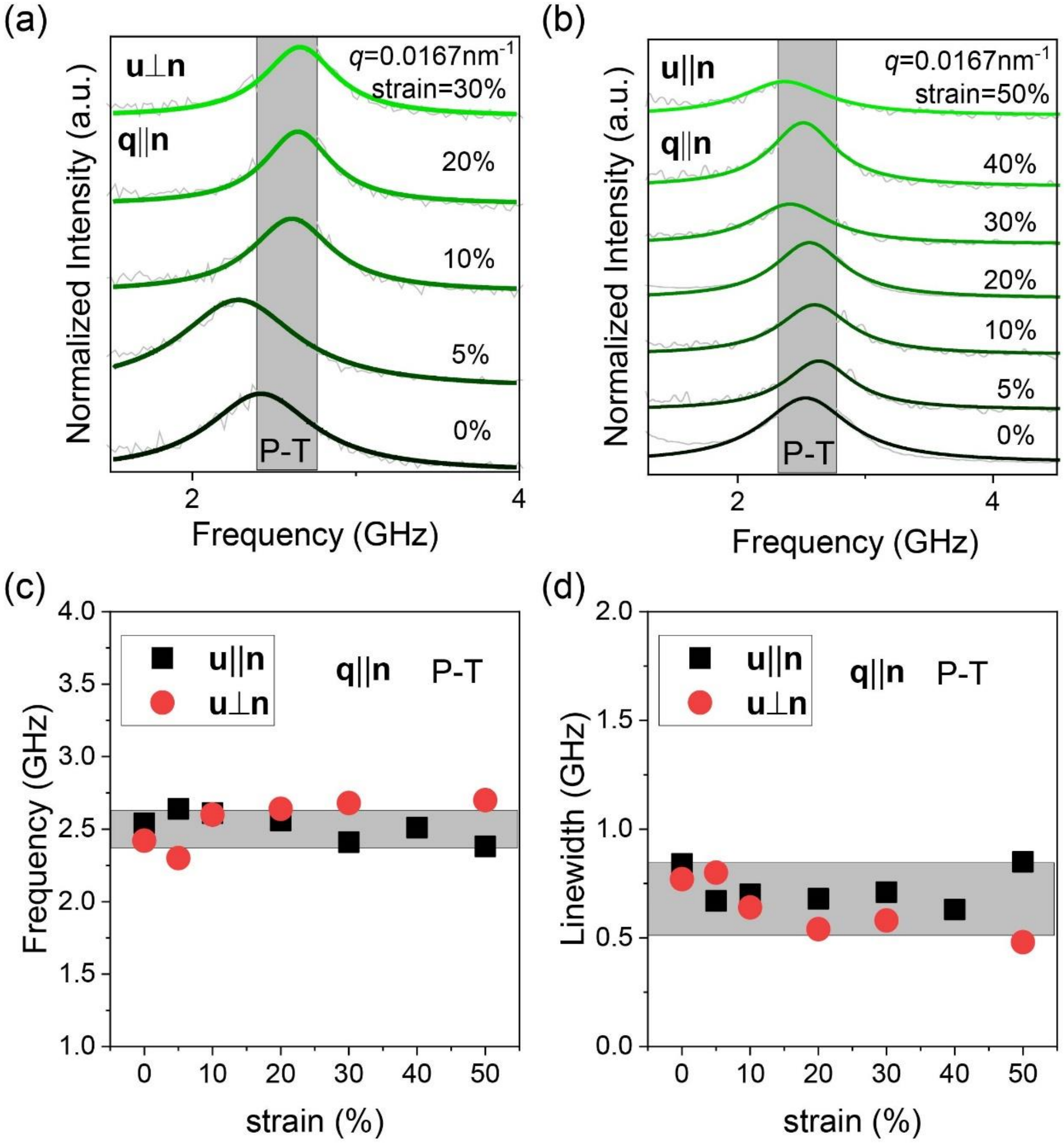

Supplementary Figure 8. Evolution of pure transverse mode on strain for monodomain LCE. At a given q=0.0167 nm^{-1} which is parallel to the director **n**, strain-dependent depolarized (VH) BLS spectra under uniaxial stretching being (a) normal and (b) parallel to the director **n**, respectively. The single peak is assigned to pure transverse (P-T) phonon. The corresponding frequency and linewidth of P-T modes is shown in (c) and (d), respectively. The BLS spectra is represented by Lorentzian shapes (colored lines). The shaded area denotes the invariant of the physical quantities.

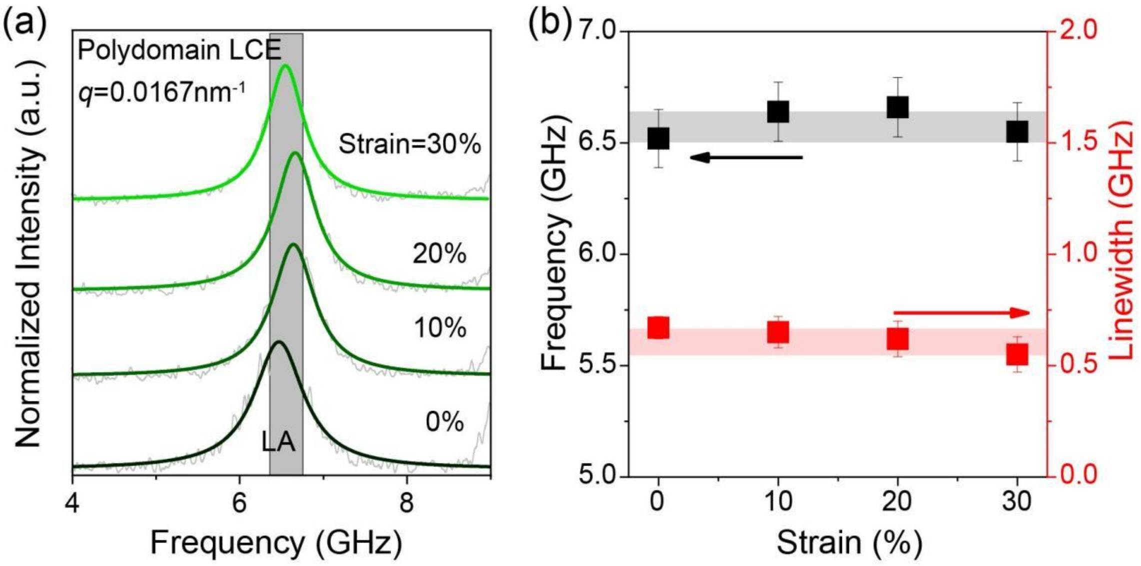

Supplementary Figure 9. Evolution of longitudinal acoustic mode on strain for polydomain LCE. (a) Polarized (VV) BLS spectra of polydomain LCE under strains at a given q=0.0167 nm^{-1}. The single peak is assigned to longitudinal acoustic (LA) phonon of which frequency and linewidth is constant with strains in (b). The BLS spectra is represented by Lorentzian shapes (colored lines). The shaded area denotes the invariant of the physical quantities.

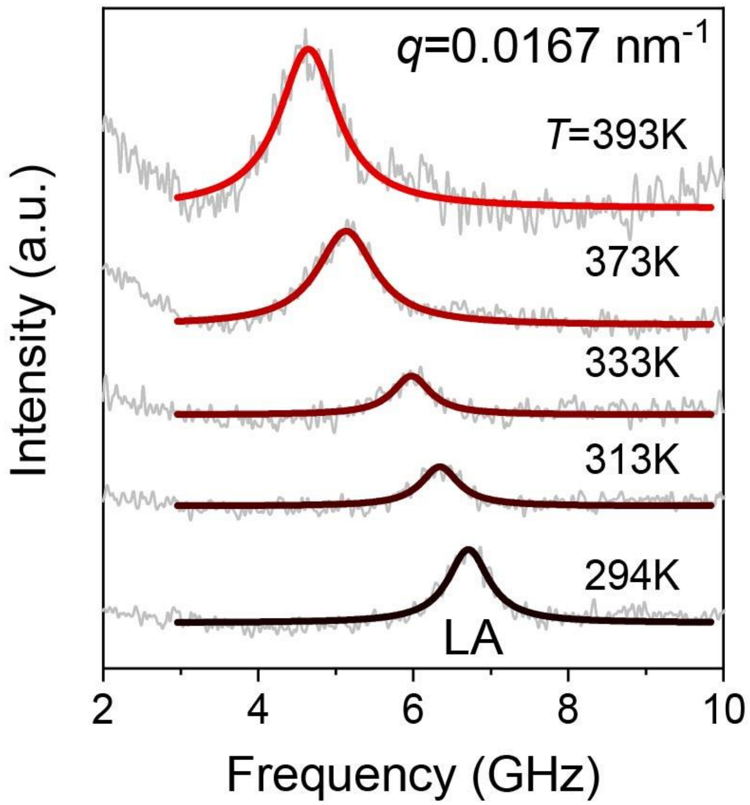

Supplementary Figure 10. Temperature-dependent polarized BLS spectra for the polydomain LCE. At a given q=0.0167 nm^{-1}, the single peak is assigned to longitudinal acoustic (LA) phonon. The BLS spectra is represented by Lorentzian shapes (colored lines).

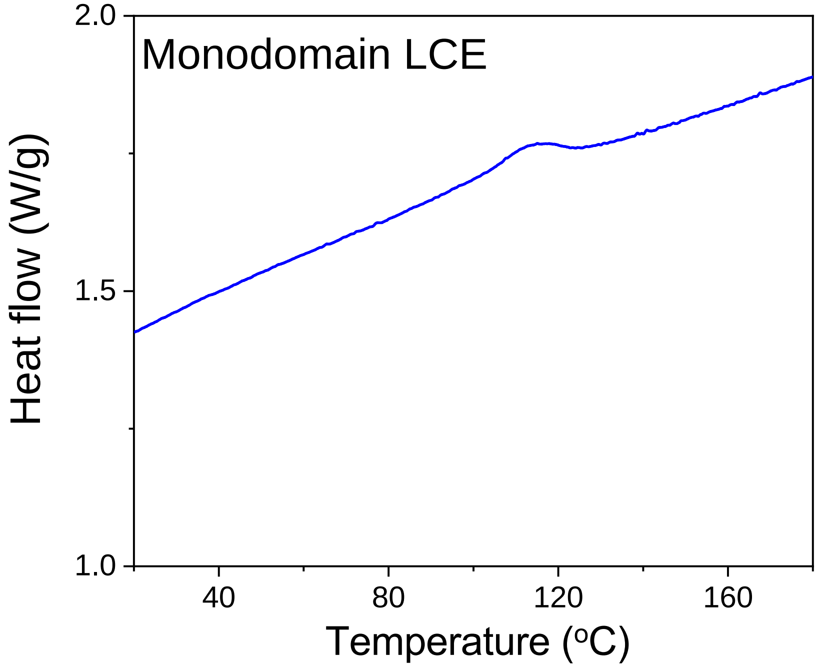

Supplementary Figure 11. Differential scanning calorimetry (DSC) curve for monodomain LCE. The DSC curve, obtained at a heating rate of 10 °C/min, shows the nematic-isotropic transition temperature at T = ~390 K.

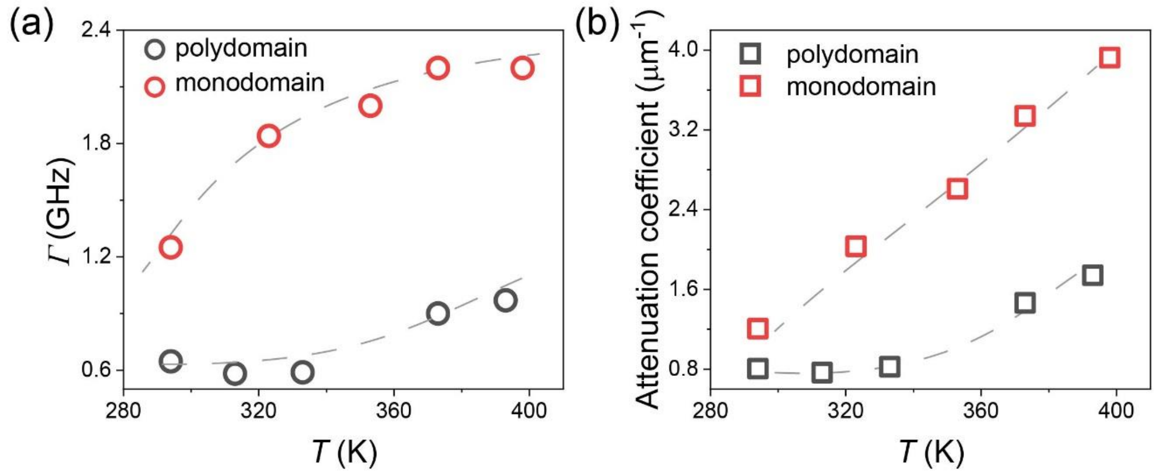

Supplementary Figure 12. Temperature-dependent viscosity. (a) Linewidth and (b) attenuation coefficient $\mu(=\pi\Gamma/c)$ of Q-L and LA phonon in Fig. 4b as a function of temperature. The dashed lines are used to guide the eyes.

Supplementary References

1 Wang, Y. *et al.* Repeatable and Reprogrammable Shape Morphing from Photoresponsive Gold Nanorod/Liquid Crystal Elastomers. *Advanced Materials* **32**, e2004270 (2020).

2 Mistry, D., Morgan, P. B., Clamp, J. H. & Gleeson, H. F. New insights into the nature of semi-soft elasticity and "mechanical-Fréedericksz transitions" in liquid crystal elastomers. *Soft Matter* **14**, 1301-1310 (2018).

3 Raistrick, T., Zhang, Z., Mistry, D., Mattsson, J. & Gleeson, H. F. Understanding the physics of the auxetic response in a liquid crystal elastomer. *Physical Review Research* **3**, 023191 (2021).
